# Surgical outcomes of anterior column reconstruction for spinal fractures caused by minor trauma-preoperative examination of the number of intervertebral bone bridges is key to obtaining good bone fusion-

**DOI:** 10.1186/s12891-024-07326-z

**Published:** 2024-03-14

**Authors:** Mitsuru Furukawa, Kanehiro Fujiyoshi, Keita Kajikawa, Yoshiomi Kobayashi, Tsunehiko Konomi, Yoshiyuki Yato

**Affiliations:** 1 Department of Orthopedic Surgery, NHO Murayama Medical Center, Tokyo, Japan; 2 Institute of Murayama Medical Center, 2-37-11 Gakuen, Musashimurayamashi, Tokyo, 208-0011 Japan

**Keywords:** Diffuse idiopathic skeletal hyperostosis, Femur proximal bone mineral density, Anterior spinal fusion, maxVB

## Abstract

**Background:**

To achieve good bone fusion in anterior column reconstruction for vertebral fractures, not only bone mineral density (BMD) and bone metabolism markers but also lever arms due to bone bridging between vertebral bodies should be evaluated. However, until now, no lever arm index has been devised. Therefore, we believe that the maximum number of vertebral bodies that are bony and cross-linked with the contiguous adjacent vertebrae (maxVB) can be used as a measure for lever arms. The purpose of this study is to investigate the surgical outcomes of anterior column reconstruction for spinal fractures and to determine the effect of bone bridging between vertebral bodies on the rate of bone fusion using the maxVB as an indicator of the length of the lever arm.

**Methods:**

The clinical data of 81 patients who underwent anterior column reconstruction for spinal fracture between 2014 and 2022 were evaluated. The bone fusion rate, back pain score, between the maxVB = 0 and the maxVB ≥ 2 patients were adjusted for confounding factors (age, smoking history, diabetes mellitus history, BMD, osteoporosis drugs, surgical technique, number of fixed vertebrae, materials used for the anterior props, etc.) and analysed with multivariate or multiple regression analyses. The bone healing rate and incidence of postoperative back pain were compared among the three groups (maxVB = 0, 2$\leqq$maxVB$\leqq$8, maxVB ≧ 9) and divided by the maxVB after adjusting for confounding factors.

**Results:**

Patients with a maxVB ≥ 2 had a significantly higher bone fusion rate (*p* < 0.01) and postoperative back pain score (*p* < 0.01) than those with a maxVB = 0. Among the three groups, the bone fusion rate and back pain score were significantly higher in the 2$\leqq$maxVB$\leqq$8 group (*p* = 0.01, *p* < 0.01).

**Conclusions:**

Examination of the maxVB as an indicator of the use of a lever arm is beneficial for anterior column reconstruction for vertebral fractures. Patients with no intervertebral bone bridging or a high number of bone bridges are in more need of measures to promote bone fusion than patients with a moderate number of bone bridges are.

## Background

A wide variety of surgical methods, including percutaneous posterior fixation, vertebroplasty, and balloon kyphoplasty, have been developed for treating osteoporotic vertebral fractures in elderly individuals [1, 2]. Anterior column reconstruction is the procedure of choice for patients with severe vertebral fractures, but osteoporosis and DM have been found to cause bone fusion failure in many cases [3–11]. On the other hand, as typified by diffuse idiopathic skeletal hyperostosis (DISH), minor trauma is even more likely to cause vertebral fractures due to stress on the lever arm caused by bone bridging between vertebrae than osteoporosis is [12–14]. Even in such cases, if the bone defect in the anterior vertebral body is large, anterior column reconstruction is necessary [15]. Bone mineral density (BMD) and bone metabolism markers are indicators of osteoporosis, but there are no lever-arm indicators. We propose that the maximum number of vertebral bodies that are bony cross-linked with contiguous adjacent vertebrae (maxVB) without interruption is a measure of the lever arm and that the greater the maxVB, the higher the fracture risk [16]. Additionally, the fracture types associated with vertebral fractures are more likely to be A3 or A4 when the maxVB = 0, B1 or B2 when the maxVB ranges from 2 to 8, and B3 or C when the maxVB is 9 or higher [17]. The maxVB is therefore considered a reasonable index for lever arms. The number of bone cross-links between vertebral bodies increases with age and is different in each individual. Thus, the incidence of DISH also increases with age, reaching more than 30% by age 70 [18, 19]. However, even when adjusted for age, there were differences in BMD and bone metabolic markers between individuals with no cross-links, few cross-links, and several cross-links. Conversely, bone cross-linking between vertebrae is not only an indicator of the lever arm but also of BMD and bone metabolism [20, 21]. Therefore, the purpose of this study is to investigate the surgical outcomes of anterior column reconstruction for spinal fractures and to determine the effect of bone bridging between vertebral bodies on the rate of bone fusion using the maxVB as an indicator of the length of the lever arm.

## Methods

**Fig. 1:**
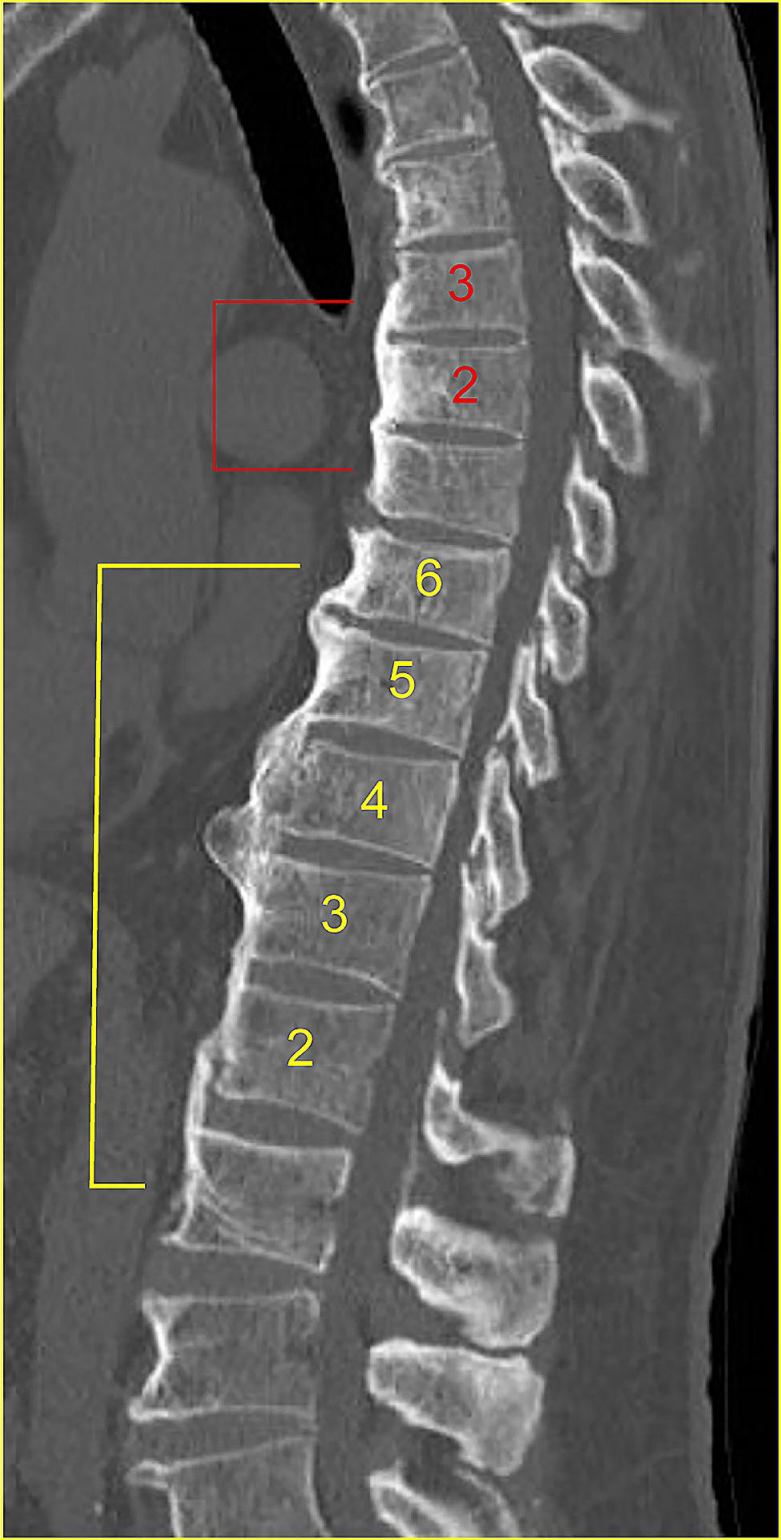
Calculation of maxVB. Sagittal CT image spanning the thoracic vertebra to the lumbar vertebra showing continuous bony bridges between adjacent vertebrae at vertebrae 3 and 6. The maximum number of contiguous bone bridges between adjacent vertebrae was defined as the maxVB, which was 6. Abbreviations: maxVB, maximum number of vertebral bodies that are bony cross-linked with contiguous adjacent vertebrae

This was a retrospective cohort study. From 2014 to 2022, 102 patients underwent anterior column reconstruction at our institution for spine fractures in the thoracic to lumbar spine range. The inclusion criteria were age 55 years or older, fractures due to minor trauma and followed for 6 months after surgery. The patient must have had a preoperative CT scan (Discovery CT 750HD) of the thoracic to lumbar spine and an X-ray and CT scan of the surgical site immediately after surgery and within 6 months to 1 year postoperatively. Eighty-one patients met the above criteria and were included in this study. The following items were examined: age at the time of fracture, sex, history of smoking and diabetes history, BMD (g/cm^2^), maxVB, number of patients per maxVB, surgical procedures, number of fixed intervertebral vertebrae, type of osteoporosis medication, surgical time (min), volume of blood loss (ml), bone fusion rate, postoperative back pain score, Barthel index, local angle before (°), immediately after surgery, 6 months after surgery (°), correction angle (°), and correction loss angle (°) [22, 23]. The maxVB = 0 (without bony bridge, *N* = 46) and the maxVB ≥ 2 (with maxVB values from 2 to 17, *N* = 35) groups were compared with respect to the above items before adjustment. The bone fusion rate, postoperative back pain score, Barthel index, and correction loss angle were compared between patients with a maxVB = 0 and those with a maxVB ≥ 2 adjusted for age, sex, fracture types, smoking history, DM history, femur proximal BMD, osteoporosis drugs, surgical technique, number of fixed vertebrae, and materials used for the anterior props. Bone fusion rate and postoperative back pain scores were compared among the three groups: maxVB = 0, maxVB (2–8; group with maxVB values from 2 to 8, *N* = 21), and maxVB (9–17; group with maxVB values from 9 to 17, *N* = 14), with maxVB = 0 serving as the reference, adjusted for the same confounders. Age at the time of surgery, DM history, and smoking history were recorded on the medical questionnaire at the initial visit. Postoperative back pain scores and Barthel indices were obtained at the postoperative visit and by telephone interview. BMD was assessed by the total proximal femur because the lumbar spine cannot be accurately measured in the presence of bony bridging (GE Medical Systems Lunar) [24]. The maxVB was defined as the maximum number of consecutive bone bridges between the adjacent vertebra from the thoracic to the lumbar spine (Fig. 1). The maxVB was calculated from the thoracic vertebra to the sacrum and was assessed by three orthopaedic surgeons. The surgical procedure was performed via the retroperitoneal space by using the anterolateral approach; curettage of the fractured vertebral body; and reconstruction with an iliac bone graft or expandable cage. Thereafter, the screws were inserted unilaterally into the side of the vertebral body and fixed with a rod (method A). Otherwise, the pedicle screw was inserted from the posterior side and fixed with a rod (P method). Method A was mainly applied from 2014 to 2017, and Method P was applied from 2018 to 2022; these methods were not selected by case. The medications for osteoporosis were selected from among bone formation stimulants (parathyroid hormone), bone resorption inhibitors (bisphosphonate, an anti-receptor activator of nuclear factor-kappa B ligand antibody, and a selective oestrogen receptor modulator), and others (romosozumab and 1,25 dihydroxyvitamin D3). Bone fusion was determined via sagittal and coronal CT scans, which revealed bone bridging between the vertebral bodies and the presence of bone fusion between the autograft bone or expandable cages and the upper and lower vertebral bodies [24–26]. In the present study, the presence of bone fusion on both cephalocaudal sides was defined as good bone fusion (for vertebrae with residual endplates, lateral fusion of the remaining vertebrae and the grafted bone was also considered fusion), while the presence of bone fusion on only one side or the absence of bilateral bone fusion was defined as poor bone fusion. Postoperative back pain was assessed using the Japanese Orthopaedic Association (JOA) scale with the following scores: none (3 points), frequently mild pain (2 points), occasional severe pain (1 point), and frequent or continuous severe pain (0 points) [27]. The local angle was determined by calculating the Cobb angle between the upper border of the upper normal vertebra and the lower border of the lower normal vertebra on lateral radiography [28, 29]. Corrective angle (°) = local angle immediately after surgery - preoperative local angle. Correction loss angle (°) = postoperative 6 M local angle - immediate postoperative local angle.

### Statistical analysis

The Statistical Package for the Social Sciences software version 26 (IBM Corp., Armonk, NY) and the statistical software R-4.0.3 (Index of /src/base/R-4 [r-project.org]) were used for all the statistical analyses. The missing values were BMD in six patients with maxVB = 0 and three patients with maxVB ≥ 2, surgical duration in six patients with maxVB = 0 and three patients with maxVB ≥ 2, and volume of blood loss in six patients with maxVB = 0 and seven patients with maxVB ≥ 2. A maxVB = 0 and a maxVB ≥ 2 were compared with the Wilcoxon rank-sum test or Fisher’s exact test. Multivariate and multiple regression analyses were performed adjusting for confounding factors, namely, the bone fusion rate, postoperative back pain score, Barthel index, and correction loss angle. *P* values < 0.05* and < 0.001** were considered to indicate statistical significance.

## Results

The means and standard deviations for all the items studied in all the patients included in this study are listed in Table 1. Compared with the maxVB ≥ 2 and maxVB = 0 before adjustment, the maxVB ≥ 2 group had a significantly higher bone union rate, Barthel index, postoperative back pain score, preoperative local angle, immediate postoperative local angle, and postoperative 6 M local angle (Table 2). The two groups did not significantly differ in terms of BMD or correction angle; however, the BMD of the group with a maxVB = 0 was more likely to be lower than that of the group with a maxVB ≥ 2, and the correction angle of the group with a maxVB = 0 was more likely to be higher than that of the group with a maxVB ≥ 2 (*P* = 0.051 and 0.056, respectively). After adjustment, the maxVB ≥ 2 group had a significantly higher bone fusion rate at 6 months after surgery (*p* < 0.001**) and postoperative back pain score (*p* < 0.001**) than the maxVB = 0 group (Tables 3 and 4). In contrast, the two groups did not significantly differ in terms of the Barthel index (*p* = 0.12) or correction loss angle (*p* = 0.3) after adjustment. When the bone healing rates and postoperative back pain scores were compared among the three groups divided by maxVB values after adjusting for confounding factors, the bone healing rate (*p* = 0.001**) and back pain score (*p* < 0.001**) were significantly higher in the maxVB (2–8) group (Tables 5 and 6).

**Table 1:** Characteristics of patients who underwent anterior strut reconstruction

Characteristics	
Age	76.1 (8.4)
Sex	
Male	25
Female	56
Smoking history	
No	72
Yes	9
DM history	
No	60
Yes	21
Femur proximal BMD (g/cm^2^)	0.54 (0.07)
maxVB	3.1 (4.5)
Number of cases per maxVB	0 (*n* = 46), 2 (*n* = 3), 3 (*n* = 4), 4 (*n* = 2), 5 (*n* = 7), 6 (*n* = 5), 9 (*n* = 2), 10 (*n* = 2), 11 (*n* = 4), 12 (*n* = 2), 13 (*n* = 2), 15 (*n* = 1), and 17 (*n* = 1)
Osteoporosis drugs	
None	32
Bone formation stimulants	35
Bone resorption inhibitors	11
Others	3
Surgical techniques	
Unilateral side screw fixation	37
Pedicle screw fixation	44
Number of fixed vertebrae	3.2 (1.5)
Materials used for the anterior props	
Iliac bone	72
Expandable cage	9
Surgical duration (min)	256 (89)
Volume of blood loss (ml)	428 (367)
Postoperative bone union	
None	20
Unilateral	19
Bilateral	42
Barthel Index	88.4 (7.5)
Postoperative back pain score	1.9 (1)
Preoperative local angle (°)	−8.5 (14)
Postoperative local angle (°)	−4 (12.7)
Postoperative local angle at 6 months (°)	−10.9 (16)
Corrective angle (°)	4.4 (9.8)
Loss of the correction angle (°)	6.8 (8.3)
Mean (SD)	

Abbreviations

DM, diabetes mellitus; BMD, bone mineral density; maxVB, maximum number of vertebral bodies that are bony cross-linked with contiguous adjacent vertebrae; SD, standard deviation

**Table 2:** Characteristics of patients who underwent anterior strut reconstruction in the maxVB = 0 and maxVB ≥ 2 groups

Characteristics	maxVB = 0 group, *n* = 46	maxVB ≥ 2 group, *n* = 35	*p* value
Age	76 (8)	76 (10)	0.5
Sex			0.15
Male	11 (24%)	14 (40%)	
Female	35 (76%)	21 (60%)	
Smoking history			0.9
No	41 (89%)	31 (89%)	
Yes	5 (11%)	4 (11%)	
DM history			0.4
No	36 (78%)	24 (69%)	
Yes	10 (22%)	11 (31%)	
Femur proximal BMD (g/cm^2^)	0.52 (0.08)	0.56 (0.07)	0.051
maxVB	0 (0)	7.3 (4.1)	< 0.001**
Osteoporosis drugs			0.2
None	16 (35%)	16 (46%)	
Bone formation stimulants	23 (50%)	12 (34%)	
Bone resorption inhibitors	4 (8.7%)	7 (20%)	
Others	3 (6.5%)	0 (0%)	
Surgical techniques			0.3
Unilateral side screw fixation	24 (52%)	13 (37%)	
Pedicle screw	22 (48%)	22 (63%)	
Number of fixed vertebrae	2.98 (1.44)	3.51 (1.56)	0.092
Materials used for the anterior props			0.4
Iliac bone	40	32	
Expandable cage	6	3	
Surgical duration (min)	253 (86)	261 (94)	0.8
Volume of blood loss (ml)	458 (423)	386 (277)	> 0.9
Postoperative bone union			< 0.001**
None	19 (41%)	1 (2.9%)	
Unilateral	14 (30%)	5 (14%)	
Bilateral	13 (28%)	29 (83%)	
Barthel Index	86 (7)	91 (7)	< 0.001**
Postoperative back pain score	1.5 (1)	2.3 (0.85)	< 0.001**
Preoperative local angle (°)	−13 (13)	−2 (14)	< 0.001**
Postoperative local angle (°)	−7 (11)	0 (14)	0.02*
Postoperative local angle at 6 months (°)	−16 (16)	−5 (14)	0.003**
Corrective angle	6 (11)	2 (8)	0.056
Loss of the correction angle (°)	9 (10)	4 (5)	0.075
Mean (SD), n (%)			
Wilcoxon rank-sum test and Fisher’s exact test			

*P* values < 0.05* and < 0.001** were considered to indicate statistical significance

Abbreviations

DM, diabetes mellitus; BMD, bone mineral density; min, minutes; maxVB, maximum number of vertebral bodies that are bony cross-linked with contiguous adjacent vertebrae; SD, standard deviation

**Table 3:** Characteristics of the maxVB = 0 and maxVB ≥ 2 groups adjusted for the confounding factors of bone fusion

Characteristics	Beta	95% CI	*p* value
maxVB = 0	—	—	
maxVB ≥ 2	1	0.47–1.5	< 0.001**
Age	0.01	−0.02–0.04	0.6
Sex	−0.28	−0.84–0.29	0.3
Fracture types			
A1	—	—	
A3	−0.27	−1.4–0.83	0.6
A4	−0.38	−1.5–0.73	0.5
B1	−1.8	−4.0–0.37	0.1
B3	−0.46	−1.8–0.83	0.5
Smoking history	−0.76	−1.5–−0.02	0.4
DM history	0.07	−0.46–0.60	0.8
Femur proximal BMD (g/cm^2^)	−0.25	−3.7–3.2	0.9
Osteoporosis drugs			
None	—	—	
Bone formation stimulants	−0.31	−0.87–0.25	0.3
Bone resorption inhibitors	−0.2	−1.0–0.58	0.6
Others	0.28	−1.2–1.8	0.7
Surgical techniques			
Unilateral side screw fixation	—	—	
Pedicle screw	0.87	0.24–1.5	0.08
Number of fixed vertebrae	−0.28	−0.48–−0.08	0.08
Materials used for the anterior props			
Iliac bone	—	—	
Expandable cage	−0.22	−0.95–0.51	0.6

*P* values < 0.05* and < 0.001** were considered to indicate statistical significance

Abbreviations

DM, diabetes mellitus; BMD, bone mineral density; min, minutes; maxVB, maximum number of vertebral bodies that are bony cross-linked with contiguous adjacent vertebrae; CI, confidence interval

**Table 4:** Characteristics of the maxVB = 0 and maxVB ≥ 2 groups adjusted for the confounding factor of postoperative back pain score

Characteristics	OR	95% CI	*p* value
maxVB = 0	—	—	
maxVB ≥ 2	19.1	5.27–87.7	< 0.001**
Age	0.94	0.85–1.02	0.14
Smoking history	0.98	0.14–7.37	> 0.9
DM history	0.53	0.11–2.28	0.4
Femur proximal BMD (g/cm^2^)	2.25	0.00–26.6	0.9
Osteoporosis drugs			
None	—	—	
Bone formation stimulants	0.39	0.08–1.80	0.2
Bone resorption inhibitors	1.03	0.12–9.70	> 0.9
Others	0.62	0.01–12.1	0.8
Surgical techniques			
Unilateral side screw fixation	—	—	
Pedicle screw	0.99	0.18–5.43	> 0.9
Number of fixed vertebrae	1.11	0.61–1.96	0.7
Materials used for the anterior props			
Iliac bone	—	—	
Expandable cage	1.9	0.30–12.5	0.5

*P* values < 0.05* and < 0.001** were considered to indicate statistical significance

Abbreviations

DM, diabetes mellitus; BMD, bone mineral density; maxVB, maximum number of vertebral bodies that are bony cross-linked with contiguous adjacent vertebrae; CI, confidence interval

**Table 5:** Characteristics of the maxVB = 0, maxVB (2–8) and maxVB (9–17) groups adjusted for the confounding factors of bone fusion

Characteristic	OR	95% CI	*p* value
maxVB = 0	—	—	
maxVB (2–8)	13	2.7–62	0.001**
maxVB (9–17)	8.1	0.59–111	0.1
Age	0.93	0.85–1	0.1
Sex	0.81	0.14–4.7	0.8
Fracture types			
A1	—	—	
A3	0.29	0.01–8.8	0.5
A4	0.44	0.017–1	0.6
B1	0.57	0	1
B3	0.33	0	1
Smoking history	0.57	0.12–2.8	0.5
DM history	0.94	0.13–6.7	0.9
Femur proximal BMD (g/cm^2^)	1	0–23,680	1
Osteoporosis drugs			
None	—	—	
Bone formation stimulants	0.32	0.064–1.6	0.2
Bone resorption inhibitors	0.88	0.081–9.7	0.9
Others	0.62	0.022–18	0.8
Surgical techniques			
Unilateral side screw fixation	—	—	
Pedicle screw	0.91	0.15–5.5	0.9
Number of fixed vertebrae	1.000	0.53–1.9	1
Materials used for anterior props			
Iliac bone	—	—	
Expandable cage	2.5	0.33–17	0.4

*P* values < 0.05* and < 0.001** were considered to indicate statistical significance

Abbreviations

DM, diabetes mellitus; BMD, bone mineral density; maxVB, maximum number of vertebral bodies that are bony cross-linked with contiguous adjacent vertebrae; CI, confidence interval

**Table 6:** Characteristics of the maxVB = 0, maxVB (2–8) and maxVB (9–17) groups adjusted for the confounding factor of postoperative back pain score

Characteristic	Beta	95% CI	*p* value
maxVB			
maxVB = 0	—	—	
maxVB (2–8)	1	0.49–1.6	< 0.001**
maxVB (9–17)	0.78	-0.19–1.7	0.1
Age	0.01	-0.017–0.037	0.5
Sex	-0.29	-0.87–0.28	0.3
Fracture types			
A1	—	—	
A3	-0.4	-1.6–0.77	0.5
A4	-0.43	-1.6–0.71	0.5
B1	-1.9	-4.1–0.3	0.09
B3	-0.36	-1.7–1	0.6
Smoking history	0.086	-0.44–0.62	0.8
DM history	-0.78	-1.5–-0.04	0.4
Femur proximal BMD (g/cm^2^)	0.14	-3.3–3.6	0.9
Osteoporosis drugs			
None	—	—	
Bone formation stimulants	-0.32	-0.88–0.25	0.3
Bone resorption inhibitors	-0.24	-1–0.56	0.6
Others	0.64	-0.57–1.9	0.3
Surgical techniques			
Unilateral side screw fixation	—	—	
Pedicle screw	0.84	0.2–1.5	0.011*
Number of fixed vertebrae	-0.27	-0.47–-0.062	0.012*
Materials used for anterior props			
Iliac bone	—	—	
Expandable cage	-0.25	-0.98–0.48	0.5

*P* values < 0.05* and < 0.001** were considered to indicate statistical significance

Abbreviations

DM, diabetes mellitus; BMD, bone mineral density; maxVB, maximum number of vertebral bodies that are bony cross-linked with contiguous adjacent vertebrae; CI, confidence interval

## Discussion

**The validity of the various factors that were considered necessary to adjust for the relationship between the lever arm (maxVB) and bone healing was also assessed.**

Bone fusion is dependent on osteogenic capacity. However, with age, bone healing is more likely to fail due to reduced osteogenic function [30, 31]. Smoking inhibits bone formation, and diabetes is associated with decreased bone quality and bone density, both of which can negatively affect bone healing [11, 32]. Osteoporosis drugs are not necessarily beneficial for bone healing. In fact, teriparatide increases bone formation capacity, and bisphosphonates increase the risk of pseudoarthrosis [33–36]. However, in this study, there were no significant differences between the maxVB = 0 and the maxVB ≥ 2 groups with respect to age, history of smoking or diabetes, or type of osteoporosis medication used. The load applied to the spine at the time of fracture is determined primarily by a person’s height, weight, muscle strength, and the work or movement performed but is also affected by other factors, such as spinal curvature and disc deterioration. For this reason, it is difficult to define the forces applied at the time of fracture [36]. In general, in patients with a large, displaced fracture or in patients where the fracture is difficult to realign, the fracture is fixed across many vertebrae at the discretion of the surgeon. Torque values are expected to differ depending on the direction of screw insertion into the vertebral body, and although the torque values for the P-method have been reported, those for the A-method have not been reported; therefore, a comparison of torque values could not be made [37]. Based on the above, the analysis was performed after adjustment of the number of fixed vertebrae and the direction of screw insertion. Finally, a previous study comparing autograft bone and mesh cages showed no difference in bone fusion [38]. In contrast, a paper comparing modern anterior instrumentation reported that the Kaneda device was better for torsion movements [39]. An analysis was performed in the current study after adjusting for autograft bone using iliac bone and expandable cages. Based on these results, confounding factors were eliminated to the maximum extent possible in this study.

**Causes of differences in bone fusion rates after anterior column reconstruction per maxVB, including case presentation and countermeasures.**

Although larger bone formation marker values indicate better bone fusion, bone formation marker values increase immediately after fracture and do not decrease for 1 year. Therefore, assessment after fracture does not provide accurate values [40–42]. BMD is currently the best indicator of bone fusion in anterior column reconstruction for spinal fracture [10]. In this study, patients with a maxVB ≥ 2 were more likely to have a higher femur bone density than those with a maxVB = 0 (*p* = 0.051) were. It has been reported that the femoral BMD in male nonfractured patients is greater when the maxVB is 2–8 than when the maxVB is 9-17 [20]. Therefore, the maxVB (2–8) can be associated with good bone healing in terms of bone density. In contrast, in patients without fracture, the maxVB, a measure of lever arm fracture extent, is positively correlated with N-propeptide of type I procollagen (P1NP), the osteogenic marker [21, 43]. Inose et al. developed the bone turnover ratio (BTR), which is a measure of bone remodelling for posterior lumbar intervertebral fusion because low P1NP and high tartrate-resistant acid phosphatase 5b (TRACP 5b) levels are risk factors for poor bone remodelling [44]. Moreover, maxVB (9–17) is favourable for bone healing in terms of osteogenic potential, as it is negatively correlated with BTR (TRACP 5b/P1NP) [21]. When bone healing during anterior strut reconstruction was adjusted for confounding factors, the maxVB = 0 group exhibited poor bone healing. This could be due to low bone density and osteogenic potential despite the absence of lever arms. Here, a patient with no bony bridging of the vertebral body (maxVB = 0) was included.

**Fig. 2:**
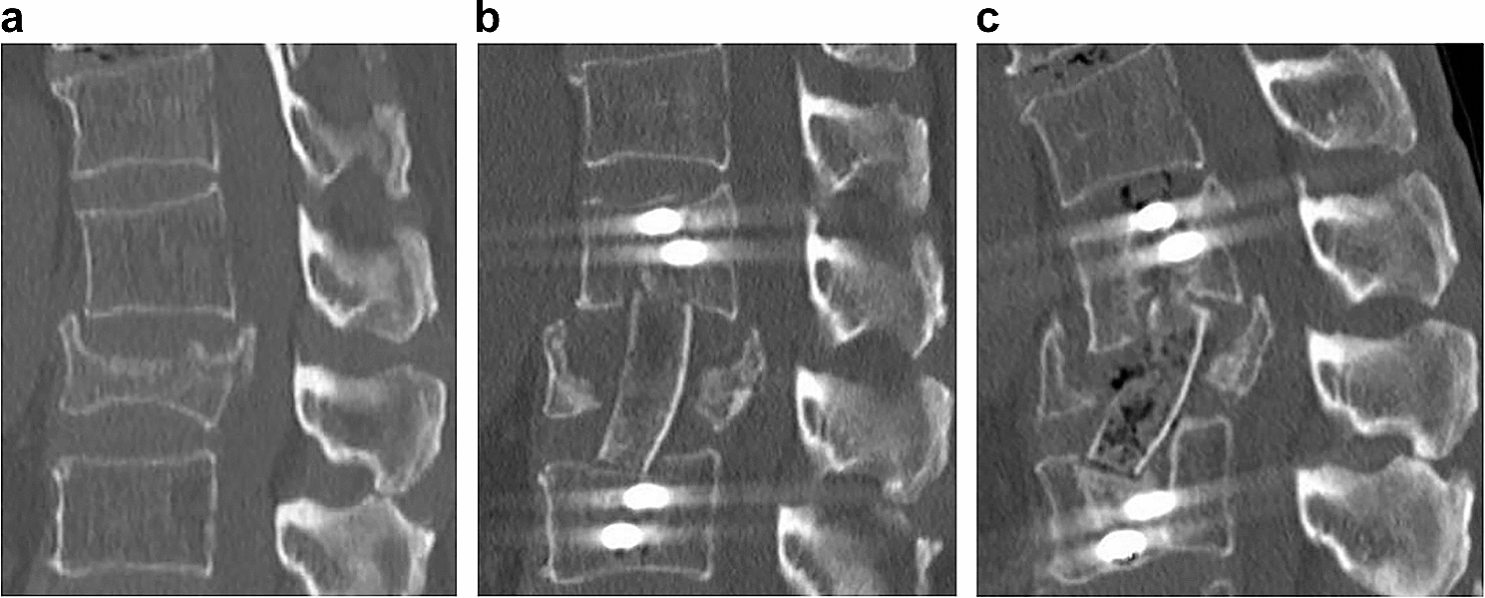
CT image reconstruction of the anterior column in a spinal fracture patient with maxVB = 0. **a** A 76-year-old male sustained an A3-type vertebral fracture at L2 after coughing. **b** The anterior column was reconstructed with the iliac bone and the L1-L3 vertebral body was fixed with unilateral side screws and a rod. **c** No bony fusion was achieved postoperatively

A 76-year-old male experienced back pain after a severe sneeze. He visited his family doctor and was diagnosed with a type A3 L2 vertebral fracture (Fig. 2a). Two months of wearing a lumbar brace and using parathyroid hormone did not relieve his back pain, so he underwent surgery at our hospital. There was no history of diabetes or smoking. The proximal femur bone density was 0.584 g/cm [2], and the maxVB was 0. The patient underwent reconstruction of the anterior column with the iliac bone and unilateral screw and rod fixation of L1-L3 (Fig. 2b). The operation time was 218 min, the blood loss volume was 443 ml, and the postoperative back pain score was 2. The immediate postoperative CT showed that the iliac bone was in contact with the cephalocaudal vertebral body, but 11 months after surgery, no bony fusion had been achieved (Fig. 2c).

**Fig. 3:**
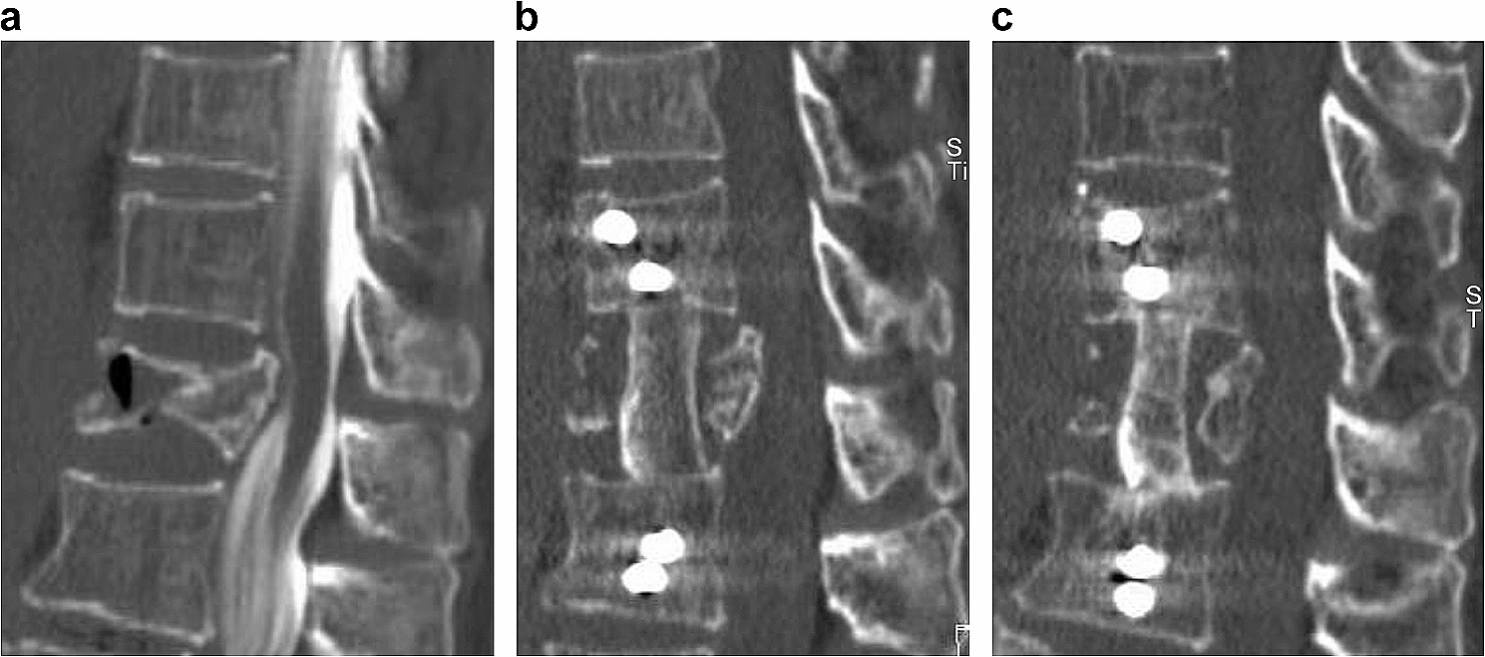
CT image reconstruction of the anterior column of a spinal fracture patient with maxVB = 6. **a** A 73-year-old female sustained an A4-type vertebral fracture at L2 after lifting a heavy object. **b** The anterior column was reconstructed with the iliac bone and the L1-L3 vertebral body was fixed with unilateral side screws and rods. **c** Postoperative CT showed bony fusion between the cephalocaudal side of the grafted bone and the remaining vertebra

When the maxVB ≥ 2 was analysed in 2 groups by dividing the maxVB (2–8) and the maxVB (9–17), it was clear that bone healing was better for the maxVB (2–8). The relatively small lever arm, high bone density, and moderately high P1NP may have promoted bone fusion in the maxVB (2–8). A 73-year-old female with a moderate number of bone cross-links (maxVB 2–8) is presented. She came to our hospital because of low back pain after lifting a heavy object. CT showed an A4-type vertebral fracture at L2 (Fig. 3a). The proximal femur bone density was 0.592 g/cm [2], and the maximum VB was 6. Reconstruction of the anterior column with the iliac bone and unilateral side screw and rod fixation of L1-L3 were performed (Fig. 3b). The operation time was 283 min, the blood loss volume was 397 ml, and the postoperative back pain score was 2. The immediate postoperative CT showed that the iliac bone was in contact with the cephalocaudal vertebral body, and 7 months postoperative CT showed that bony fusion had been achieved between the cephalocaudal side of the grafted bone and the remaining vertebrae (Fig. 3c).

Compared with maxVB (2–8), which is disadvantageous for bone healing, maxVB (9–17) was considered to have higher osteogenic potential but lower bone density and larger lever arms. Although we cannot conclude whether there is a significant difference in bone healing due to the small number of patients, it is suggested that further extension of the fixation range and stabilization of the fracture site may result in good bone healing. A patient with a high number of bone cross-links (maxVB = 17) is presented.

**Fig. 4:**
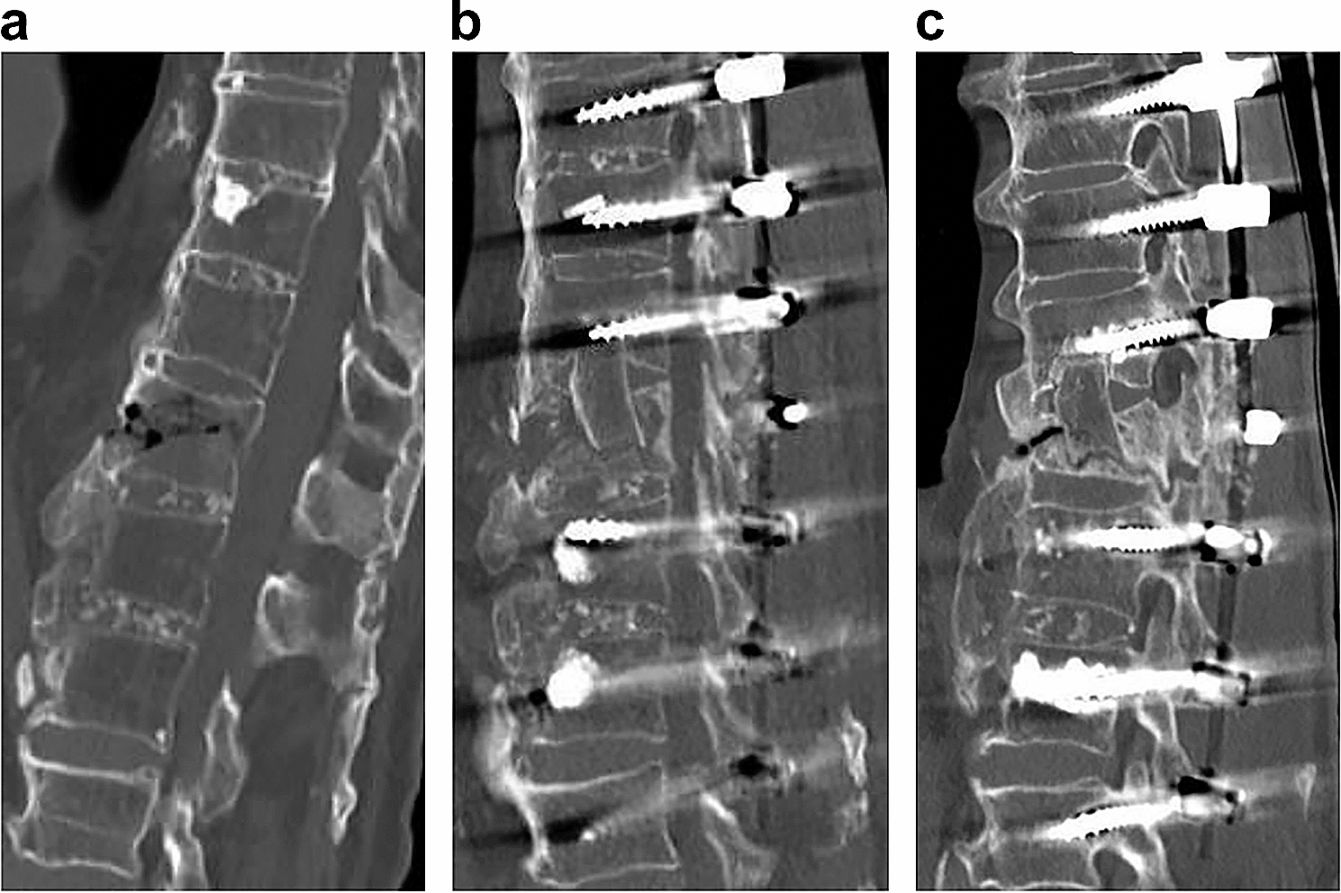
CT image reconstruction of the anterior column of a spinal fracture patient with maxVB = 17. **a** An 80-year-old male sustained a reverse chance fracture at the T12 level during a fall. **b** Anterior column reconstruction with iliac bone and T10-L2 pedicle screw fixation were performed. **c** Postoperative CT showed bony fusion between the cephalad side and lateral side of the grafted bone and the remaining vertebra

An 80-year-old male came to our hospital complaining of back pain after a fall, but no neurological symptoms were observed. He had no history of diabetes or smoking. On CT, the patient had a proximal femur density of 0.588 g/cm [2], a maxVB = 17, and a reverse chance of fracture at T12 (Fig. 4a). Anterior post reconstruction with iliac bone and T10-L2 pedicle screw fixation were performed (Fig. 4b). The operation time was 532 min, the blood loss volume was 618 ml, and the postoperative back pain score was 3. Immediately postoperative CT showed that the iliac bone was in contact with the cephalocaudal vertebrae, and 8 months postoperative CT showed that bony fusion had been achieved between the cephalad side and lateral side of the grafted bone and the remaining vertebrae (Fig. 4c).

Therefore, when performing anterior strut reconstruction for spine fractures, patients without bone bridging should be aggressively treated for osteoporosis because of poor bone fusion despite the absence of lever arms. Additionally, in some cases of maxVB (9–17), a long fixation range should be considered, accounting for the long lever arm.

**The participants’ daily life (Barthel index) and back pain scores were reported after anterior column reconstruction by maxVB.**

Generally, type B and type C fractures are high-energy traumas. These fractures must be carefully managed because spinal cord injury can occur if such fractures are missed and progressively dislocated [13, 14]. However, it was reported that the comparison between a maxVB = 0 and a maxVB ≥ 2 in patients with type B and C fractures did not change the degree of preoperative paralysis or the rate of improvement in postoperative paralysis [7]. Furthermore, the postoperative Barthel index did not differ between the two groups. Early detection of vertebral fracture and timely surgery to prevent severe paralysis may have contributed to the postoperative Barthel index. There was no difference in clinical outcomes or visual analogue scale scores between patients who underwent short-segment pedicle screw fixation for spinal fractures with and without postoperative pseudoarthrosis, according to a previous report [45]. In contrast, some reports have shown that postoperative pseudoarticulation can cause back pain, and some patients are forced to undergo revision surgery [46–48]. The maxVB = 0 group had a lower postoperative back pain score, and bone fusion was worse in the maxVB = 0 group than in the maxVB ≥ 2 group. Surprisingly, there was no significant difference in the correction loss angle between the two groups, suggesting that low back pain due to kyphosis deformity is unlikely. Furthermore, a comparison of the three groups revealed that maxVB (2–8) was associated with the mildest back pain. As mentioned in the section on bone healing, high bone density and the fact that the lever arm is not very long may contribute to the degree of postoperative back pain, as the fracture site is easily stabilized and bone healing is easily achieved by anterior column reconstruction. Improved bone fusion and longer range fixation across the fracture site may be the key to reducing postoperative back pain, as described in the section on bone fusion.

## Limitations

This study has several limitations. That is, the study was retrospective in nature, and the number of patients evaluated was small. Bone cross-linking develops with increasing age, and the number of bone cross-links is also affected by degeneration. However, the severity of back pain before fracture was not investigated. Furthermore, the surgical procedure was not randomized. Future prospective studies should be conducted to enhance the validity of this study.

## Conclusions

It is useful to use the maxVB as an indicator of the lever arm when performing anterior column reconstruction for spinal fractures. Compared with patients with a maxVB ≥ 2, patients with a maxVB = 0 had poorer bone healing and more postoperative back pain. According to our 3-group comparison, maxVB (2–8) resulted in better bone healing and less postoperative back pain. The results of this study suggest that in patients with a maxVB = 0 and no bone bridging between vertebrae, bone metabolism may have an effect on the failure of bone fusion, and osteoporosis should be treated carefully. In addition, in patients with a maxVB ≥ 9 and long lever arms, it is important to stabilize the fracture site by lengthening the range of fixation.

## Data Availability

The data is generated in this study is included in the manuscript.

## Abbreviations

DM: Diabetes mellitus
BMD: Bone mineral density
maxVB: Maximum number of vertebral bodies that are bony cross-linked with contiguous adjacent vertebrae
SD: Standard deviation
P1NP: N-propeptide of type I procollagen
CT: Computed tomography
TRACP 5b: Tartrate-resistant acid phosphatase 5b
